# Effects of Pre-Oxidation on Haloacetonitrile and Trichloronitromethane Formation during Subsequent Chlorination of Nitrogenous Organic Compounds

**DOI:** 10.3390/ijerph17031046

**Published:** 2020-02-07

**Authors:** Ao Wang, Chenshuo Lin, Zhen Shen, Zhigang Liu, Hang Xu, Jiapei Cheng, Xin Wen

**Affiliations:** 1 Key Laboratory of Integrated Regulation and Resource Development on Shallow Lakes, Ministry of Education, College of Environment, Hohai University, Nanjing 210098, China; 1714040327@hhu.edu.cn (A.W.); lincs23@126.com (C.L.); 160205010001@hhu.edu.cn (Z.S.); 191305010002@hhu.edu.cn (J.C.); 181305010009@hhu.edu.cn (X.W.); 2 Wanjiang University of Technology, Maanshan 243031, China; 3 Ningbo Water Supply Co., Ltd, No. 348 Xinhe Road, Ningbo 315041, China; zcigmondliu@126.com

**Keywords:** amino acids, nitrogenous disinfection by-products, pre-oxidation, bromide, toxicity

## Abstract

The reaction between organic matter and disinfectants leads to the formation of disinfection byproducts (DBPs) in drinking water. With the improvement of detection technology and in-depth research, more than 1000 kinds of DBPs have been detected in drinking water. Nitrogenous DBPs (N-DBPs) are more genotoxic and cytotoxic than the regulated DBPs. The main methods are enhanced coagulation, pretreatment, and depth technologies which based are on conventional technology. Amino acids (AAs) are widely found in surface waters and play an important role by providing precursors from which toxic nitrogenous disinfection by-products (N-DBPs) are generated in chlorinated drinking water. The formation of N-DBPs, including dichloroacetonitrile, trichloroacetonitrile, and trichloronitromethane (TCNM), was investigated by analyzing chlorinated water using ozone (OZ), permanganate (PM), and ferrate (Fe(VI)) pre-oxidation processes. This paper has considered the control of pre-oxidation over N-DBPs formation of AAs, OZ, PM, and Fe(VI) pre-oxidation reduced the haloacetonitrile formation in the downstream chlorination. PM pre-oxidation decreased the TCNM formation during the subsequent chlorination, while Fe(VI) pre-oxidation had no significant influence on the TCNM formation, and OZ pre-oxidation increased the formation. OZ pre-oxidation formed the lowest degree of bromine substitution during subsequent chlorination of aspartic acid in the presence of bromide. Among the three oxidants, PM pre-oxidation was expected to be the best choice for reducing the estimated genotoxicity and cytotoxicity of the sum of the measured haloacetonitriles (HANs) and TCNM without bromide. Fe(VI) pre-oxidation had the best performance in the presence of bromide.

## 1. Introduction

Chlorine is the most widely used disinfectant because of its significant role in inactivating microorganisms in drinking water and protecting human health from diseases. Chlorine is effective, cheap, and remains active in distribution systems for a considerable period of time. However, the major concern regarding using chlorine is the formation of potentially carcinogenic disinfection by-products (DBPs), such as trihalomethanes, haloacetic acids, nitrosamines, cyanogen halides, haloacetonitriles (HANs), haloacetamides, and halonitromethanes (HNMs), through reactions between precursor materials and chlorine [1,2,3,4]. Recently, reports of nitrogenous DBPs (N-DBPs) increased rapidly [5,6,7,8,9,10,11]. HANs, including dichloroacetonitrile (DCAN), trichloroacetonitrile (TCAN), bromochloroacetonitrile (BCAN), and dibromoacetonitrile (DBAN) are often detected in chlorinated water [12]. Trichloronitromethane (TCNM) is the most important HNM that is often identified in chlorinated water [13,14]. 

Nitrogenous organic compounds (NOC) play an important role in providing precursors to generate N-DBPs, such as HANs and HNMs, during chlorination [15,16]. Amino acids (AAs) are the most studied subclass of NOC; they are found widely in fresh waters and are difficult to remove using traditional drinking water treatment processes [17]. In natural river water, the concentration of total dissolved AAs was found to be 100–500 μg/L, and yet it can rise to 300–6000 μg/L in eutrophic lakes [18]. Aspartic acid (Asp) and tryptophan (Trp) were shown to produce DCAN of 6%–9% conversion yields, and histidine (His) and tyrosine (Tyr) also exhibited very high amount of HAN formation during chlorination [15,19,20]. In previous studies, the formation of TCNM during the chlorination of AAs was surveyed. The yields were from Trp, asparagine, and Tyr at 2.99, 1.05, and 1.00 μmol/mmol, respectively [21]. Yang et al. evaluated TCNM formation during the chlorination of 20 AAs at pH 7.2 and found that the yields of TCNM were less than that of DCAN [22]. 

Pre-oxidation is an effective strategy to reduce the formation of DBPs before chlorination disinfection [23,24]. Ozone (OZ), permanganate (PM), and ferrate (Fe(VI)) are the most widely used in pre-oxidation link in Chinese water treatment technology. Ozone (OZ) can partially oxidize NOCs, including DBP precursors, and decrease DBP formation from subsequent chlorination. Previous studies have revealed that OZ could generally decrease the yields of HANs but increase the formation of TCNM [25,26,27]. Permanganate (PM) pre-oxidation increased the removal of DBP precursors by subsequent conventional water treatment processes and could effectively remove the precursors of DCAN (28.6%) and TCNM (35.7%) [28]. Chu et al. evaluated the impact of PM pre-oxidation on DCAN formation from the chlorination of microcystin-LR. The DCAN formation during chlorination decreased regardless of the PM dose after 30 min pre-oxidation, yet the yields of DCAN were not significantly different when the concentration of PM increased from 1.0 to 5.0 mg/L [7]. Ferrate (Fe(VI)) pre-oxidation was proposed as an alternative pre-oxidant in drinking water treatment processes because it produced little or no hazardous by-products [29]. Although many researchers have studied the removal of C-DBPs and N-DBPs in recent years, few studies have considered the control of pre-oxidation over N-DBPs formation of AAs, especially with the presence of bromine in aqueous water.

The overarching objective of our research was to elucidate HAN and TCNM formation from AAs and from three pre-oxidants–OZ, PM, and Fe(VI). The main aims of our study were: (1) to determine the formation of HANs and TCNM potential precursors from the specified four AAs and compare the effectiveness of pre-oxidation for the control of different N-DBP classes, (2) to propose the formation pathways of HANs and TCNM from chlorination of AAs, (3) to assess the impact of bromide on the formation of HAN and TCNM during pre-oxidation subsequent chlorination, and (4) to estimate the toxicity of N-DBPs in different scenarios. 

## 2. Materials and Methods

### 2.1. Materials and Chemicals

Table S1 presents the qualities of water used in our experiments. The water was collected from Jiaokou Reservoir, which is the source of drinking water in Ningbo City, Zhejiang Province, China. Four AAs, including Trp, Tyr, Asp, and His were intentionally selected as precursors based on their charge property, polarity, and hydrophobicity. The physicochemical characteristics and structures of the selected AAs are listed in Table 1.

Three AAs (Trp, Tyr, His) were supplied by a certified vendor (Sigma-Aldrich) and Asp was purchased from Wako (Osaka, Japan). DCAN, TCAN, BCAN, DBAN, and TCNM were obtained from Sigma-Aldrich (St. Louis, Missouri, USA). All the solutions were prepared in distilled water produced by a Millipore Milli-Q Gradient water purification system (Billerica, MA, USA) and all bottles used for the experiment were pre-washed and dried at 105 °C for 24 h in the dark. Four AA solutions, as well as free chlorine stock solutions (1000 mg/L) prepared from sodium hypochlorite (~5%) (Sinopharm Chemical Reagent Co., Ltd., Shanghai, China), were freshly prepared prior to use. They were stored in a glass-stoppered flask covered with aluminum foil. Bromide ion solutions were prepared by using diluted sodium bromide solution (NaBr, GR grade). The saturated OZ stock solution (20 mg/L) was freshly prepared by using an ozone generator (Model CF-G-3-10; Guolin, Qingdao, China) fed with continuous ultra-high-purity oxygen gas. Fe(VI) was prepared as solid potassium ferrate (K_2_FeO_4_) of 99% purity (Battle Corporation) by a method based upon the oxidation of ferric nitrate with hypochlorite [30]. Working solutions of Fe(VI) (1 g/L as Fe) were generated by adding solid K_2_FeO_4_ to sodium hydroxide solution, and were used within 30 min of their preparation. All other materials were of at least analytical purity and purchased from Sinopharm Chemical Reagent Co., Ltd. (Shanghai, China), unless otherwise mentioned.

### 2.2. Experimental Procedures 

Three repetitions were made for each experiment. For all experiments during chlorination without pre-oxidants, four AA sample solutions diluted to 0.1 mM were buffered at pH 7.0 using 4 mM sodium bicarbonate and adjusted with 1 M HCl or NaOH. Chlorination was conducted in 500 mL chlorine demand-free glass-stoppered bottle. The disinfectant/AA molar ratio was 30:1 providing sufficient disinfection during the whole reaction procedure. All chlorinated samples were stored headspace-free in the dark at room temperature (~22 °C). After reacting for certain incubation time, the tested samples were quenched and extracted immediately with methyl tert-butyl ether, based on the US EPA Method 551.1. Furthermore, an orthogonal matrix experiment was designed to examine the impact of pre-oxidation on DCAN, TCAN, and TCNM formation. In this experiment, the applied OZ, PM, and Fe(VI) concentrations were 0, 1, 2, and 5 mg/L, respectively, and were added to AAs solutions. These samples were mixed on a stir plate for 30 min. After 30 min of contact time, the previously prepared chlorine solution was added to the samples. HANs and TCNM were measured with a 24 h incubation time at 22 ± 1 °C and pH 7 ± 0.1. All samples were prepared in triplicate, and the error bars indicate the standard deviation of replicate measurements (*n* = 3). 

### 2.3. Analytical Methods

The N-DBPs determined for the batch scale experiment included HANs and TCNM, and were carried out using purge and trap (OI Analytical, Eclopse 4660, College Station, TX, USA) and gas chromatography/mass spectrometry (GC/MS, Shimadzu-QP-2010 Ultra, Japan) [20]. For this analysis, the detection limits for them were below 1 μg/L and the recoveries of them were 81.5–118.7%. In addition, residual and total chlorine concentrations were analyzed by a portable spectrophotometer (HACH DR 1900) based on the HACH method 8021. 

The HANs is extracted from a 50 mL tube by taking 20 mL water solution, adding 4 g anhydrous Na_2_SO_4_ and then fully mixing and dissolving, adding 2 mL MTBE extractant, shaking it by hand for 1min, then fully shaking it on a shaking table for 15 min and then leaving it for 10 min to be layered completely, and extracting the upper extractant for the detection. The HANs is separated by HP-5 (30 m × 0.25 mm × 0.1 μm) capillary column, the inlet temperature of the gaschromatograph is set to 250 °C and the split ratio is 2:1. The TCNM is detected by gas chromatography/mass spectrometry (GC/MS, Shimadzu-QP-2010 Ultra, Japan) liquid-liquid extraction. We used 50 mL tubes to measure 20 mL water solution, adding 4 g anhydrous Na_2_SO_4_ and then fully mixing and dissolving, adding 2 mL MTBE extractant, shaking it by hand for 1 min, then fully shaking it on a shaking table for 15 min and then leaving it for 10 min to be layered completely, then extracting the upper extractant for the detection of TCNM. The TCNM is separated by HP-5 (30 m × 0.25 mm × 0.1 μm) capillary column, the inlet temperature of the gaschromatograph is set to 250 °C, taking 1 μL of the sample to be tested each time, the temperature of the MS detector is set to 300 °C. Initial temperature in the furnace is maintained at 30 °C for 15 min, then heated to 160 °C and lasts 10min.The recovery rate is 97.5%, the minimum detection limit is less than 1 μg/L.

### 2.4. Determinations of Predicted Toxicity

The predicted genotoxicity of the measured DBPs (i.e., HANs and TCNM) was calculated by dividing the measured concentration of DBPs by the published genotoxicity potencies in the Chinese hamster ovary (CHO) comet assay (4 h exposure). The genotoxicity is the dose required to elicit a toxic response in 50% of the cells (Table S2), which is a unitless value [1,31]. Similarly, the predicted cytotoxicity was calculated by dividing the measured concentration of DBPs by the published LC50 values. The cytotoxicity is the dose required to induce 50% viability of the cells for CHO cells (72 h exposure) and is represented in Table S3 [1,31]. 

## 3. Results and Discussion

### 3.1. HAN and TCNM Formation during Different Times of Chlorination

**Figure 1:**
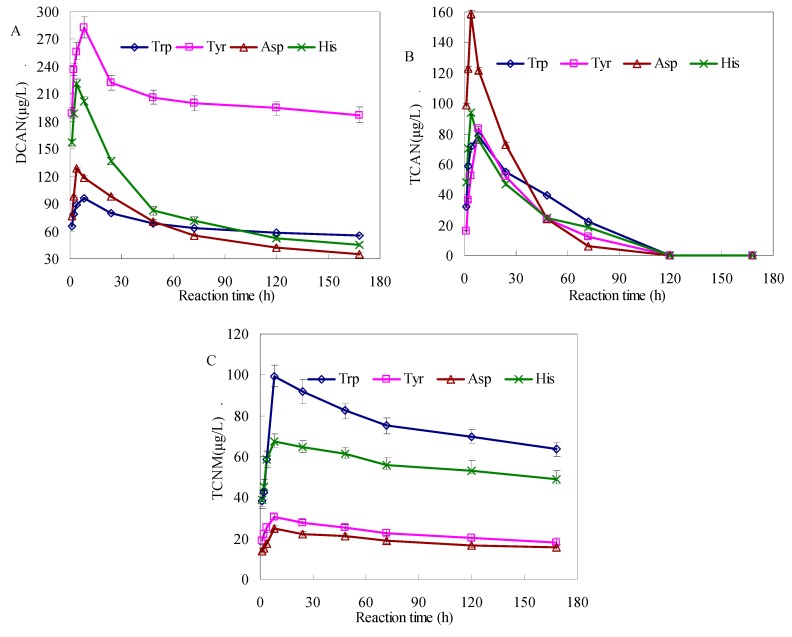
Formation of haloacetonitriles (HANs) (**A**,**B**) and trichloronitromethane (TCNM) (**C**) during chlorination of tryptophan (Trp), tyrosine (Tyr), aspartic acid (Asp), and histidine (His) at different contact times (Trp = 0.1 mM, Tyr = 0.1 mM, Asp = 0.1 mM, His = 0.1 mM, temperature = 22 ± 1 °C, pH = 7 ± 0.2, and Cl_2_ = 3 mM). The bars represent the standard deviation of replicate measurements (*n* = 3).

Figure 1 displays the time-dependent formation of HANs and TCNM during chlorination of Trp, Tyr, Asp, and His. It was found that the formation of HANs and TCNM quickly reaches the maximum at the beginning of chlorination and eventually decreases at longer periods. In the initial stage, the rapid increase of the HANs was mainly due to the reaction Asp and chlorine. Then the concentration of Asp and chlorine reduced gradually with reaction time, which might lead to the reduced generation rate of HANs. In addition, HANs is instable, with a high hydrolysis rate when the generation of HANs gradually increased. Therefore, the concentration of HANs peaked when there was an equilibrium between the formation rate of HANs and the hydrolysis rate of HANs. Asp and His produced the largest amount of DCAN at 8 h (concentrations of 128.5 and 221.4 μg/L, respectively), while the highest concentrations of DCAN from Trp and Tyr occurred at 4 h (concentrations of 95.8 and 282.4 μg/L, respectively) (Figure 1A). The maximum yields of DCAN were Tyr > His > Asp > Trp. Hong et al. reported that the chlorination of AAs could lead to nitrile formation [32], and the chlorination of nitrile by HOCl might contribute to the formation of DCAN [33]. Tyr formed the highest level of DCAN during chlorination at pH 7 because the polypeptides and hydrophobic substances with amino acid moieties were used to create more DCAN [34]. Although Gosian et al. reported that Asp produced the maximum yields of DCAN (pH = 8) during chlorination [35], it was not in agreement with our study. The different experimental conditions—hydrolysis, pH, and Cl_2_ dosage—perhaps were the main reason for DCAN formation. 

Figure 1B shows the formation of TCAN during chlorination of Trp, Tyr, Asp, and His. All AAs produced TCAN at the beginning of chlorination. For the four AAs evaluated during chlorination, the time at which the maximum concentration of TCAN occurred from the four AAs was consistent with that of DCAN. Asp generated the highest value of TCAN at 4 h (158.6 μg/L), His at 4 h (93.8 μg/L), followed by Tyr at 8 h (83.4 μg/L), and Trp at 8 h (78.2 μg/L). Apparent first-order rate constants for the decomposition of TCAN and DCAN in the presence of chlorination were 1.74 × 10^−5^ S-1 and 3.06 × 10^−6^ S-1 (pH = 7, temperature = 20 °C), respectively [36]. In general, the trend of TCAN formation with increasing contact time can be explained as follows: because of the presence of excess chlorine, Cl_2_ was a stronger oxidizing agent and thus increased the reaction yield of TCAN. Compared to DCAN and TCNM, TCAN was relatively unstable and hydrolyzed faster in the presence of chlorine [37]. Hence, TCAN was below the detection limit after 120 h.

The maximum concentrations of TCNM appeared at 8 h (99.2, 30.5, 24.8, and 67.4 μg/L) from Trp, Tyr, Asp, and His, respectively (Figure 1C). The yields of TCNM were Trp > His > Tyr > Asp for complete reaction time. Trp with R groups had a higher reactive activity benzene ring. And, it was concluded that the formation of TCNM may be because of the reaction of chlorine with a benzene ring, which gave rise to the hydrolysis of the benzene ring and contributed to the formation of TCNM. Although Tyr was also with R group, it might be the passivation of hydroxyl (–OH) groups that hindered the hydrolysis of benzene rings. In addition, 24 h was selected as the basis reaction time for subsequent experiments, considering the delivery time for water from drinking water treatment plants is generally not more than 24 h; and it has often been chosen in earlier studies [21,38]. 

### 3.2. HAN and TCNM Formation during Pre-Oxidation Subsequent Chlorination

**Figure 2:**
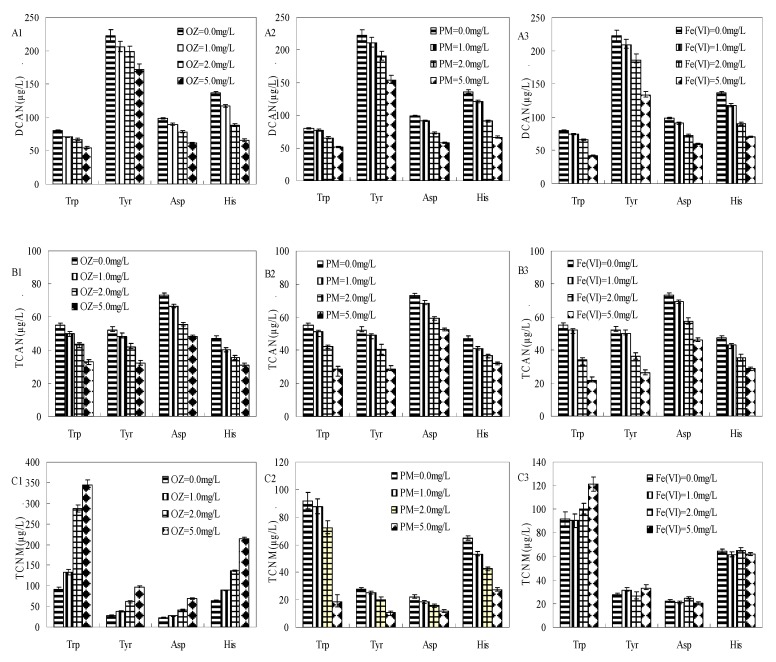
Formation of dichloroacetonitrile (DCAN) (**A1**–**A3**), trichloroacetonitrile (TCAN) (**B1**–**B3**), and TCNM (**C1**–**C3**) during pre-oxidation (ozone (OZ), permanganate (PM), and Fe(VI)) of Trp, Tyr, Asp, and His subsequent chlorination (Trp = 0.1 mM, Tyr = 0.1 mM, Asp = 0.1 mM, His = 0.1 mM, Cl2 = 3 mM, pre-oxidant contact time = 0.5 h, chlorine contact time = 24 h, pH = 7 ± 0.2, and temperature = 22 ± 1 °C). The bars represent the standard deviation of replicate measurements (*n* = 3).

The yields of DCAN and TCNM from 24 h chlorination of Trp, Tyr, Asp, and His after 30 min pre-oxidation at different OZ, Fe(VI), and PM doses (1, 2, and 5 mg/L) are shown in Figure 2. HAN and TCNM reduction rates of pre-oxidation are summarized in Table S4. Overall, increasing the dosages of the pre-oxidizing chemicals had positive effects on the decrease of HAN formation, but not on that of TCNM formation. 

As illustrated in the results for A1, A2, and A3 (Figure 2), the yields of DCAN decreased with increase in dosages of the pre-oxidizing chemicals. During subsequent chlorination, as the pre-oxidants dose increased from 0 to 5 mg/L, OZ pre-oxidation decreased the DCAN yields of Trp, Tyr, Asp, and His by 31.6%, 22.5%, 37.4%, and 52.1%, respectively. Similarly, PM pre-oxidation decreased the DCAN formation by 36.1%, 30.6%, 41.6%, and 51.0%, respectively, while Fe(VI) pre-oxidation decreased its formation by 47.1%, 40.1%, 38.9%, and 48.4%, respectively. Results for TCAN formation during 24 h post-chlorination of Trp, Tyr, Asp, and His with 30 min pre-oxidation are shown in Figure 2(B1–B3). Post-chlorination has a similar impact on the formation of TCAN with DCAN (Table S4). HANs are commonly derived from a single pathway and oxidation disrupts their formation pathway. This result is in agreement with previous reports [17,37]. 

TCNM formation during 24 h post-chlorination of AAs after 30 min pre-oxidation is depicted in C1, C2, and C3 (Figure 2). OZ pre-oxidation and subsequent chlorination had a negative effect on TCNM formation. As the pre-oxidants dose increased from 0 to 5 mg/L, OZ pre-oxidation decreased the TCNM yields of Trp, Tyr, Asp, and His by −276.4%, −250%, −210%, and −232%, respectively, during subsequent chlorination. PM pre-oxidation decreased the TCNM formation by 79.6%, 61.9%, 45.7%, and 57.8%, respectively. However, Fe(VI) pre-oxidation had no obvious influence on the formation of TCNM. Compared with PM and Fe(VI), OZ might produce more TCNM by oxidizing amine groups to the nitro group [14,24]. 

### 3.3. Proposed Formation Pathways of HAN and TCNM Formation

Formation pathway of DCAN and TCNM from chlorination of AAs was proposed by Yang et al. as shown in Figure S1 [22]. When N-terminal amino group of AAs was dechlorinated, decarboxylation coupled with chloride loss led to the formation of a chlorinated imine. Further elimination of hydrochloric acid formed a nitrile. Further, the chlorination of nitrile by HOCl might lead to the formation of DCAN and TCAN (Figure S1a). “R” side chain in AAs was an electron-withdrawing functions group and might be broken easily during chlorination. The functional group of “R”, an indole group in Trp, chlorinated phenol in Tyr, a carboxyl in Asp, and aromatic ring in His could be withdrawn easily, and then DCAN was formed during chlorination. TCAN was also formed during chlorination of these AAs because of the electron-withdrawing of their side chain “R.” The formation pathway of TCNM was also proposed, as shown in Figure S1b. A critical branching point appeared where the C = N double bond in chlorinated imine was oxidized by HOCl. Elimination of hydrochloric acid, followed by further oxidation of HOCl and elimination of H_2_O and aldehyde, formed CHCl_2_-N(OH)Cl. TCNM was formed after a reaction series of elimination, oxidation, and deprotonation. A release of the R-CHO group was proposed as a key step leading to TCNM formation. 

The formation of HANs generally depended on the relative single pathway, thus pre-oxidation might probably cut off the formation pathway of HANs [39]. The standard oxidation potential of OZ, PM, and Fe(VI) were 2.1 V, 1.7 V, and 2.2 V, respectively [24,40,41]; OZ and Fe(VI) were both more powerful oxidants than PM. However, the rank for the effect of the three pre-oxidations on HAN and TCNM formation was not in agreement with their standard oxidation potentials. It might be due to their oxidation characteristics (e.g., oxidation potential, oxidation kinetics) and could depend in part on the characteristics of DBP and AAs. More research is to be conducted to confirm this hypothesis. 

The proposed pathway of TCNM formation during OZ pre-oxidation subsequent chlorination is shown in Figure S2. Direct OZ reaction oxidized the amine of AAs to form 2-nitroacetate [14], the decarboxylation of 2-nitroacetate produced nitromethyl anion, while the carboxylic acid functional group aids nitromethane formation. TCNM was produced after the amine oxidized to a 2-nitroethanol, followed by the chlorination of 2-nitroethanol, decarboxylation and the release of CH2R’ group [42]. 

As indicated by the above discussion of the proposed pathway, HAN and TCNM formation of all the AAs during pre-oxidation subsequent chlorination was the same. Therefore, Asp was selected to further investigate the influence of bromide and estimate the toxicity of HANs and TCNM.

### 3.4. Effect of Bromide

Bromide is widespread in the water environment. The formation of brominated N-DBPs in drinking water is a very noticeable issue, because they present higher frequency of cytotoxicity and genotoxicity than their chlorinated N-DBPs.

**Figure 3:**
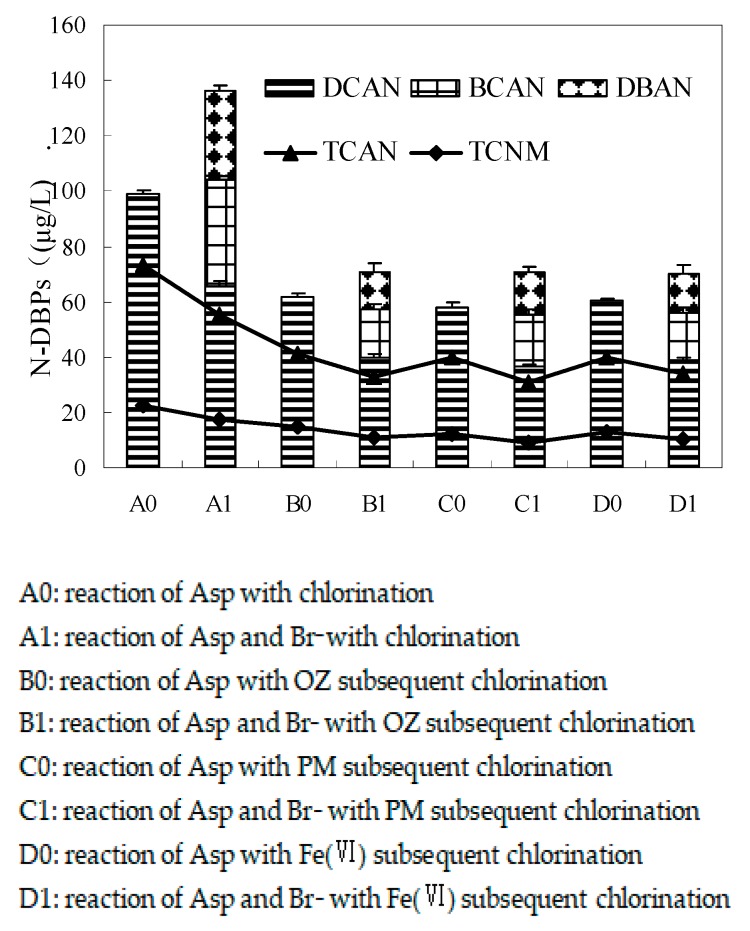
Effect of bromide on the formation of HAN and TCNM during pre-oxidation subsequent chlorination of Asp (Asp = 0.1 mM, oxidant (OZ, PM, and Fe(VI)) = 5 mg/L, Br^_^ = 1 mg/L, pre-oxidant contact time = 0.5 h, chlorine contact time = 24 h, pH = 7 ± 0.2 and temperature = 22 ± 1 °C). The bars represent the standard deviation of replicate measurements (*n* = 3).

Figure 3 illustrates the effect of bromide on the formation of HAN and TCNM during pre-oxidation subsequent chlorination of Asp. Four HANs, including DCAN, BCAN, DBAN, TCAN, and TCNM, were detected. During the chlorination of Asp, as bromide concentrations increased from 0 to 1 mg/L, the total concentrations of dihaloacetonitriles (DHANs) increased from 98.7 to 136.4 μg/L, while the concentrations of TCAN and TCNM reduced. This might be the reason why their brominated substitutions were not detected, which was in agreement with the formation of brominated DBPs during chlorination of natural organic matter in the presence of bromide [43]. The presence of bromide shifted HANs to more brominated species and increased the concentration of the total HANs because bromine species were more effective substitution agents than the corresponding chlorine species [5]. Compared to the reaction of Asp with chlorination, it had a similar impact on the formation of HANs and TCNM during pre-oxidation subsequent chlorination of Asp in the presence of bromide. 

Increasing bromide concentrations shifted the distribution of DHANs from DCAN to BCAN and to DBAN. Bromine incorporation factor (BIF) is generally used to explore the degree of bromine substitution [44]. In order to illustrate the influence of bromine on the formation of DHANs during pre-oxidation subsequent chlorination, BIF was estimated using the following equation.

(1) $$BIF\left(DHANs\right)=\frac{\left[BCAN\right]+2\left[DBAN\right]}{\left[DCAN\right]+\left[BCAN\right]+\left[DBAN\right]}$$

BIF decreased during pre-oxidation subsequent chlorination of Asp (0.46−0.53) compared with the reaction of Asp with chlorination (0.57) in the presence of bromide (1 mg/L). BIF value of OZ pre-oxidation subsequent chlorination (0.46) was lower than that of Fe(VI) pre-oxidation subsequent chlorination (0.48), but the BIF value of PM pre-oxidation subsequent chlorination (0.53) was the highest. It was clear that OZ produces lower BIF than PM and Fe(VI) pre-oxidation during subsequent chlorination of Asp, and forms the lowest degree of bromine substitution. 

### 3.5. Estimated Toxicity of HANs and TCNM in Different Pre-Oxidation Methods

Pre-oxidation reduced the HAN formation in some methods, but not all for TCNM, OZ pre-oxidation even let TCNM increase. So it is essential to give a comprehensive evaluation criterion to choose a more effective method for controlling toxicity.

**Figure 4:**
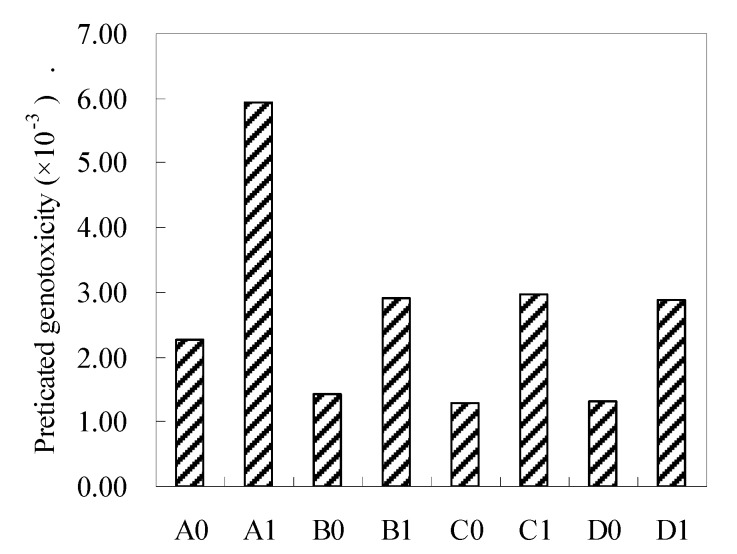
Estimated genotoxicity of the sum of the measured HAN and TCNM during pre-oxidation subsequent chlorination of Asp (The meaning of the number is the same as above, Asp = 0.1 mM, oxidant (OZ, PM, and Fe(VI)) = 5 mg/L, Br^_^ = 1 mg/L, pre-oxidant contact time = 0.5 h, chlorine contact time = 24 h, pH = 7 ± 0.2, and temperature = 22 ± 1 °C). The bars represent the standard deviation of replicate measurements (*n* = 3).

**Figure 5:**
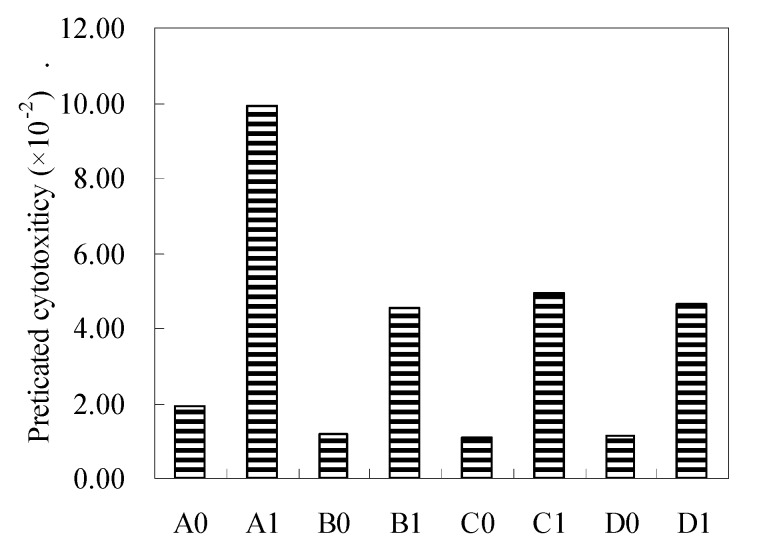
Estimated cytotoxicity of the sum of the measured HAN and TCNM during pre-oxidation subsequent chlorination of Asp (The meaning of the number is the same as above, Asp = 0.1 mM, oxidant (OZ, PM, and Fe(VI)) = 5 mg/L, Br^_^ = 1 mg/L, pre-oxidant contact time = 0.5 h, chlorine contact time = 24 h, pH = 7 ± 0.2, and temperature = 22 ± 1 °C). The bars represent the standard deviation of replicate measurements (*n* = 3).

Figure 4 and Figure 5 depict the estimated genotoxicity and cytotoxicity of the sum of the measured DBPs, including DCAN, TCAN, BCAN, DBAN, and TCNM, during pre-oxidation subsequent chlorination of Asp. It is observed that adding bromine greatly increased the estimated genotoxicity and cytotoxicity of the sum of the measured DBPs. Pre-oxidation decreased the estimated genotoxicity and cytotoxicity with/without bromine. TCNM was 1–2 orders of magnitude more genotoxic than DCAN, TCAN, and BCAN (Table S2). OZ pre-oxidation increased the TCNM yields (Figure 2), while OZ pre-oxidation increased the estimated genotoxicity of the sum of the measured DBPs compared to PM pre-oxidation and Fe(VI) pre-oxidation. DCAN, BCAN, and DBAN were 1–2 orders of magnitude more cytotoxic than TCNM, and OZ pre-oxidation could control the HAN formation better compared to PM pre-oxidation and Fe(VI) pre-oxidation. OZ pre-oxidation further reduced the estimated genotoxicity of the sum of the measured DBPs compared to PM pre-oxidation and Fe(VI) pre-oxidation. In addition, Fe(VI) pre-oxidation had no remarkable influence on the formation of TCNM, but the value of Fe(VI) pre-oxidation subsequent chlorination was lower than PM pre-oxidation subsequent chlorination. Therefore, PM pre-oxidation was expected to be a good choice for reducing the estimated genotoxicity and cytotoxicity of the sum of the measured DBPs without bromide, while Fe(VI) pre-oxidation had a better performance in the reduction of the estimated genotoxicity and cytotoxicity of the sum of the measured DBPs in the presence of bromide.

It is noteworthy that AAs cannot represent the whole precursor compound in an aqueous environment because HANs and TCNM are not unique drivers of toxicity. In authentic drinking water, other DBPs will appear and the synergistic mixture effects are also to be considered. 

## 4. Conclusions

In this study, AAs formed DCAN, TCAN, and TCNM during chlorination. It was found that Asp and His produced the largest amounts of DCAN and TCAN, which occurred at 8 h (1.68% and 2.01% of DCAN, and 1.09% and 0.64% of TCAN). However, the highest concentrations of DCAN and TCAN from Trp and Tyr occurred at 4 h (0.87% and 2.57% of DCAN, 0.54% and 0.585 of TCAN). The maximum yields of DCAN and TCAN were Tyr > His > Asp > Trp and Asp > His > Tyr > Trp, respectively. The maximum concentrations of TCNM all appeared at 8 h (99.2, 30.5, 24.8, and 67.4 μg/L from Trp, Tyr, Asp, and His, respectively). The yields of TCNM were Trp > His > Tyr > Asp. 

Experiments show that pre-oxidation reduced the HAN formation, but not all for TCNM.OZ, PM, and Fe(VI) reduced the HAN formation in the downstream chlorination. PM decreased the TCNM formation during the subsequent chlorination, and Fe(VI) had no significant influence on the TCNM formation, while OZ increased the formation of TCNM. OZ, PM, and Fe(VI) pre-oxidation decreased the value of BIF during subsequent chlorination of Asp in the presence of bromide. OZ pre-oxidation during subsequent chlorination of Asp formed the lowest degree of bromine substitution. Without the participation of bromide, PM pre-oxidation had a better performance in the reduction of controlling the estimated genotoxicity and cytotoxicity of the sum of the measured HANs and TCNM. While in the presence of bromide, Fe(VI) pre-oxidation was more effective for controlling toxicity with bromide.

## Figures and Tables

**Table 1:** Amino acids (AAs) selected for the study.

Polarity	Type	Amino Acid	Structure	pK1	pK2	pK3	Designation *
**Non-Polar**		*Tryptophane*	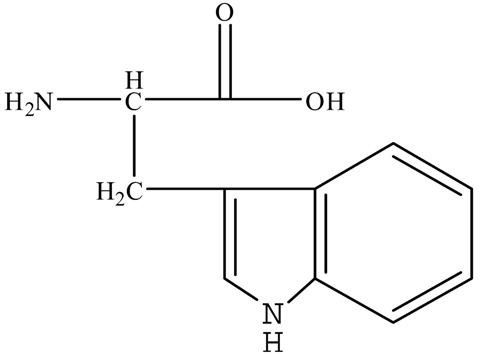	2.5	9.4	-	L
**Polar**	Neutral	Tyrosine	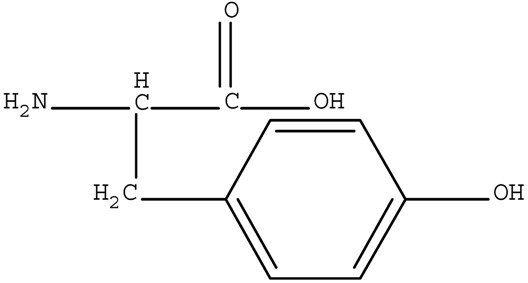	2.2	9.2	10.5	W
	Acidic	Aspartic Acid	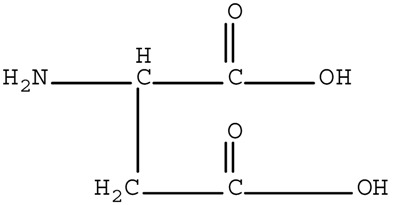	2.0	10.0	4.04	W
	Basic	Histidine	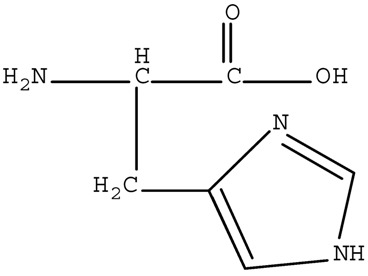	1.8	9.3	6.8	W

* Hydrophobic = L, Hydrophilic = W.

## Supplementary Materials

The following are available online at https://www.mdpi.com/1660-4601/17/3/1046/s1, Figure S1: Proposed formation pathway of DCAN and TCNM from chlorination of AAs, Figure S2: Proposed formation pathway of TCNM formation during OZ pre-oxidation subsequent chlorination, Table S1: Quality of water used in the experiment from Jiaokou reservoir, Table S2: Genotoxicity potency (single cell gel electrophoresis assay toxicity potency on CHO cells) of measured DBPs, Table S3: Cytotoxicity ( LC50† values for CHO cells) of measured DBPs, Table S4: HAN and TCNM reduction rates (%) of pre-oxidation (the dosages of the pre-oxidizing chemicals OZ, PM, and Fe(VI) were 5 mg/L).

## Abbreviations

AAS: Amino acids; N-DBPs, nitrogenous disinfection by-products; DCAN, dichloroacetonitrile; TCAN, trichloroacetonitrile; TCNM, trichloronitromethane; OZ, ozon; PM, permanganate; Fe(VI), ferrate; DBPs, disinfection by-products; HANs, haloacetonitriles; HNMs, halonitromethanes; BCAN, bromochloroacetonitrile; DBAN, dibromoacetonitrile; NOC, nitrogenous organic compounds; Asp, aspartic acid; Try, tryptophan; His, histidine; Tyr, tyrosine; DHANs, dihaloacetonitriles; GC/MS, gas chromatography/mass spectrometry; CHO, Chinese hamster ovary; BIF, bromine incorporation factor.
