# Supplementary material for: Effects of Pre-Oxidation on Haloacetonitrile and Trichloronitromethane Formation during Subsequent Chlorination of Nitrogenous Organic Compounds
Source: Int J Environ Res Public Health. 2020 Feb 7;17(3):1046. doi: 10.3390/ijerph17031046 (PMC7038144; doi:10.3390/ijerph17031046)
Supplement: Supplementary file 1 [file ijerph-17-01046-s001.pdf]

Supplementary Material

Effects of pre-oxidation on haloacetonitrile and trichloronitromethane formation during subsequent chlorination of nitrogenous organic compounds

Ao Wang[1], Chenshuo Lin[1], Zhen Shen[1,3], Zhigang Liu[2], Hang Xu[1,*], Jiapei Cheng[1] and Xin Wen[1]

[1] Key Laboratory of Integrated Regulation and Resource Development on Shallow Lakes, Ministry of Education, College of Environment, Hohai University, No.1 Xikang Road, Nanjing 210098, China; 1714040327@hhu.edu.cn(A.W.); lincs23@126.com(C.L.); 160205010001@hhu.edu.cn(Z.S.); 191305010002@hhu.edu.cn(J.C.); 181305010009@hhu.edu.cn(X.W.)

[2] Ningbo Water Supply Co., Ltd, No. 348 Xinhe Road, Ningbo 315041, China; zcigmondliu@126.com

[3] Wanjiang University of Technology, Maanshan 243031, China

* Correspondence: xuhang810826@hhu.edu.cn

Outline

Table S1. Quality of water used in the experiment from Jiaokou reservoir

Table S2. Genotoxicity potency (single cell gel electrophoresis assay toxicity potency on CHO cells) of measured DBPs

Table S3. Cytotoxicity (LC50† values for CHO cells) of measured DBPs

Table S4. HAN and TCNM reduction rates (%) of pre-oxidation

Figure S1. Proposed formation pathway of DCAN and TCNM from chlorination of AAs

Figure S2. Proposed formation pathway of TCNM formation during OZ pre-oxidation subsequent chlorination

Table S1. Quality of water used in the experiment from Jiaokou reservoir.

pH	*Temperature(°C)*	*Turbidity(NTU)*	COD_{Mn} *(mg/L)*	NH_3-N *(mg/L)*	*TN(mg/L)*
6.8	23	0.45	2.5	0.05	2.17

Table S2. Genotoxicity potency (single cell gel electrophoresis assay toxicity potency on CHO cells) of measured DBPs.

DBP	***DCAN***	***TCAN***	***TCNM***	***BCAN***	***DBAN***
Genotoxicity Potency (M)	2.75×10^{-3}	1.01×10^{-3}	9.34×10^{-5}	3.24×10^{-4}	4.71×10^{-5}

Table S3. Cytotoxicity (LC50† values for CHO cells) of measured DBPs.

DBP	***DCAN***	***TCAN***	***TCNM***	***BCAN***	***DBAN***
Cytotoxicity (M)	5.73×10^{-5}	1.60×10^{-4}	5.36×10^{-4}	8.46×10^{-6}	2.85×10^{-6}

Table S4. HAN and TCNM reduction rates (%) of pre-oxidation (the dosages of the pre-oxidizing chemicals OZ, PM, and Fe(VI) were 5 mg/L).

	DCAN				TCAN				TCNM			
	Trp	Tyr	Asp	His	Trp	Tyr	Asp	His	Trp	Tyr	Asp	His
OZ	31.6	22.5	37.4	52.1	39.7	38.4	34.3	34.1	-276.4	-250	-210	-232
PM	36.1	30.6	41.6	51.0	48.3	44.9	28.0	31.2	79.6	61.9	45.7	57.8
Fe(VI)	47.1	40.1	38.9	48.4	60.6	49.7	36.7	39.0	-32.3	-19.4	8.1	4.2

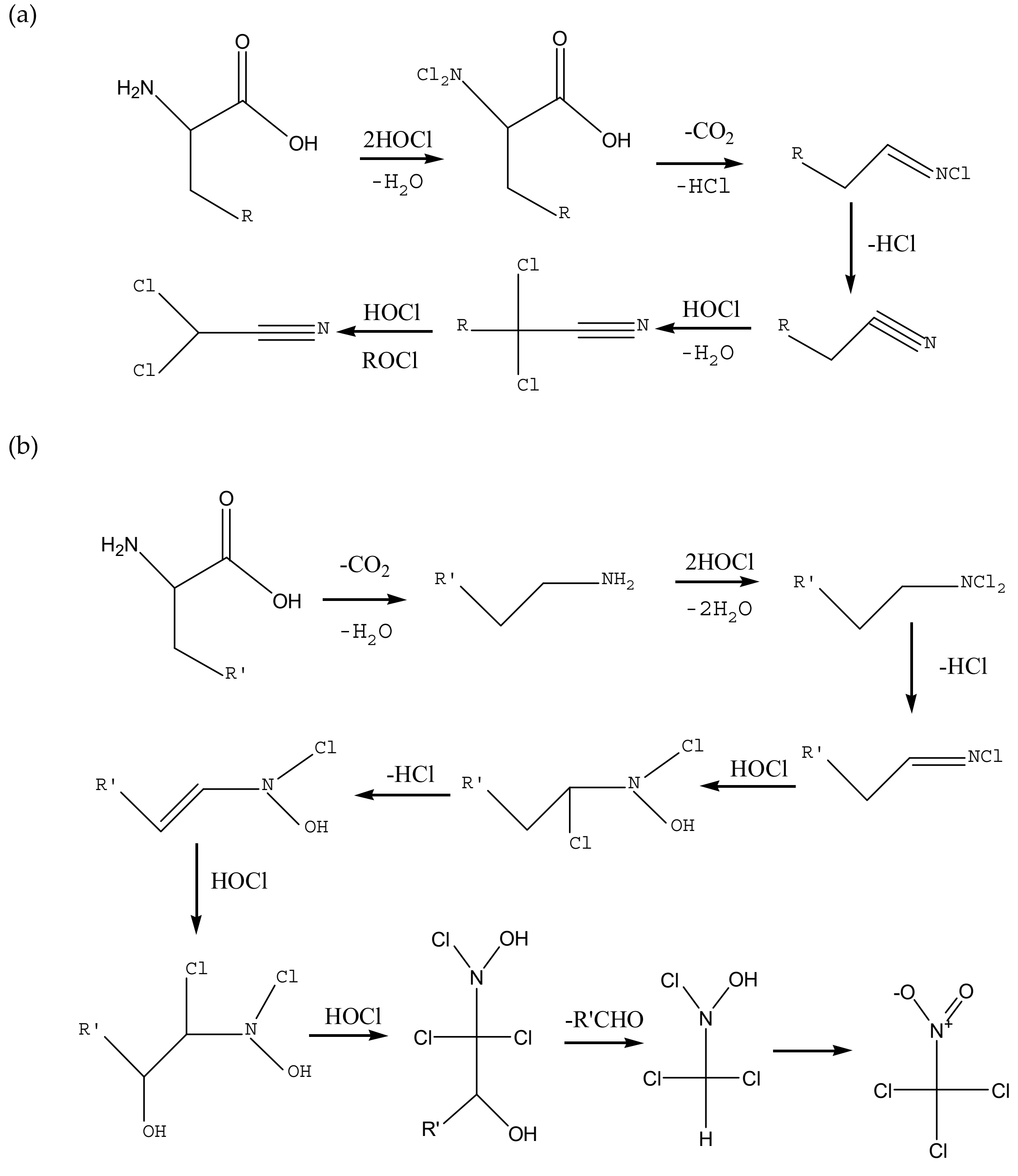

Figure S1. Proposed formation pathway of DCAN and TCNM from chlorination of AAs [1, 2].

References

[1] X. Yang, Q. Shen, W. Guo, J. Peng, Y. Liang, Precursors and nitrogen origins of trichloronitromethane and dichloroacetonitrile during chlorination/chloramination, CHEMOSPHERE, 88 (2012) 25-32.

[2] W. Chen, Z. Liu, H. Tao, H. Xu, Y. Gu, Z. Chen, J. Yu, Factors affecting the formation of nitrogenous disinfection by-products during chlorination of aspartic acid in drinking water, SCI TOTAL ENVIRON, 575 (2017) 519-524.

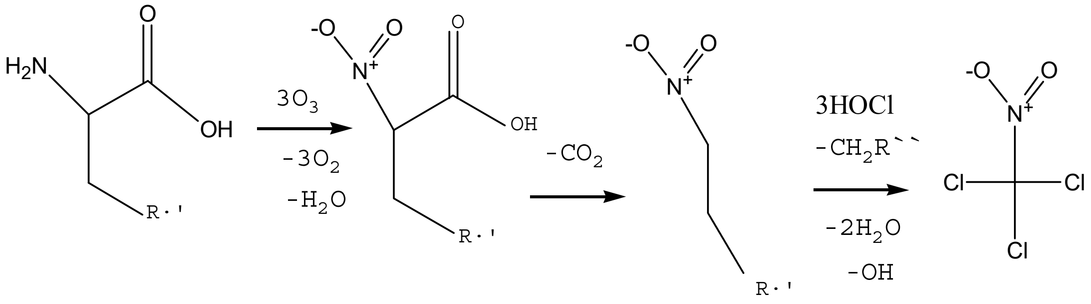

Figure S2. Proposed formation pathway of TCNM formation during OZ pre-oxidation subsequent chlorination [1, 2].

References

[1] U. von Gunten, Ozonation of drinking water: Part I. Oxidation kinetics and product formation, WATER RES, (2003) 1443-1467.

[2] D.L. Mccurry, A.N. Quay, W.A. Mitch, Ozone Promotes Chloropicrin Formation by Oxidizing Amines to Nitro Compounds, ENVIRON SCI TECHNOL, 50 (2016) 1209-1217.
